# High throughput method to determine the surface activity of antimicrobial polymeric materials

**DOI:** 10.1016/j.mex.2021.101593

**Published:** 2021-11-25

**Authors:** Wilma van Rensburg, Wikus Ernst Laubscher, Marina Rautenbach

**Affiliations:** BIOPEP^TM^ Peptide Group, Department of Biochemistry, Faculty of Science, University of Stellenbosch, South Africa

**Keywords:** Self-sterilising materials, Antimicrobial surface screening, Disk diffusion assay, JIS Z 2801, Resazurin assay

## Abstract

Surface colonization by microorganisms, combined with the rise in antibiotic resistance, is the main cause of production failures in various industries. Self-sterilising materials are deemed the best prevention of surface colonization. However, current screening methods for these sterilising materials are laborious and time-consuming. The disk diffusion antimicrobial assay and the Japanese industrial standard method for antimicrobial activity on solid surfaces, JIS Z 2801, were compared to our modified solid surface antimicrobial assay in terms of detecting the activity of antibiotic-containing cellulose disks against four bacterial pathogens. Our novel assay circumvents the long incubation times by utilising the metabolic active dye, resazurin, to shorten the time in which antibacterial results are obtained to less than 4 h. This assay allows for increased screening to identify novel sterilising materials for combatting surface colonisation.

• Disk diffusion assay could only detect the activity of small compounds that leached from the material over 20–24 h.
• JIS Z 2801 was also able to detect the surface activity of non-polar compounds, thought to be inactive based on the disk diffusion results.
• The resazurin solid surface antimicrobial assay could obtain the same results as the JIS Z 2801, within a shorter time and in a high-throughput 96-well plate setup.

## Graphical abstract

**Figure:**
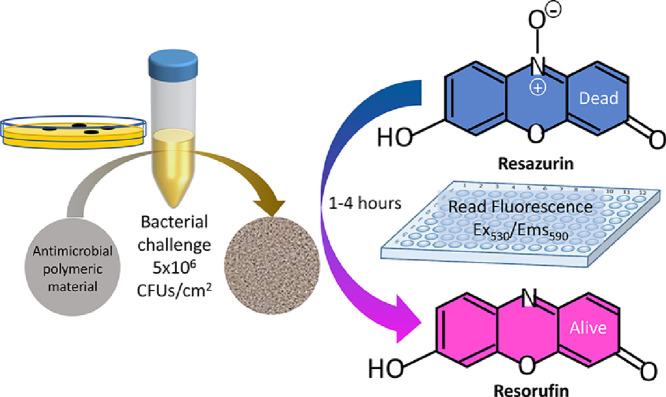

Specifications Table

**Table:** 

Subject Area:	Materials Science
More specific subject area:	Antimicrobial discovery and development
Method name:	Resazurin solid surface antimicrobial assay
Name and reference of original method:	*n/a*
Resource availability:	*n/a*

## Background

Amidst the alarming rise in resistance of microorganisms against the available antibiotics and the link between resistance and biofilm formation, much development has gone into curbing surface colonization as a preventative measure. In medicine, every surface concerning a patient, from operation surfaces to implants and catheters are at risk as a source of infection [1], [2], [3]. In agriculture, post-harvest infections and spoilage of produce [4,5], especially in the light of international export, shipping or long-term storage, whereas the food processing industry faces the greatest risk of pathogens infections with fresh and ready-to-eat foods [5], [6], [7], [8]. It is evident that from the widespread occurrence of continual infection by pathogenic organisms that the initial surface infection and subsequent surface colonization is at the core of the problem. It would therefore be prudent to try to limit the propagation of microbes on surfaces thereby preventing microbial related product losses and human infection. This can be achieved with the use of active materials that kill pathogens or prevent adhesion.

Active antimicrobial surfaces are divided into three general categories: anti-adhesion, slow-release or contact killing surfaces. Anti-adhesion materials are directly aimed at preventing surface colonization by considering surface roughness [9,10], surface hydrophobicity [11], and the target organism [11]. Slow release or contact killing surfaces are applied to industries where it is beneficial that the microorganisms that encounter the surface are killed rather than just preventing surface colonization. Informative reviews on available technologies on active surface development are available [12,13]. The research into antimicrobial or self-sterilising solid surfaces has been extensive but have been limited by screening methodologies. Reviews on available methods [14] indicate that the major limitations are time, cost in terms of consumables, and that these methods can become quite laborious.

One of the best performing methods specifically testing solid surface activity, identified in a recent comparative study [15], is the Japanese industrial standard method, JIS Z 2801. However, the well-known disk diffusion type assays are more commonly used by research groups to assess the antimicrobial activity of novel materials [16], [17], [18], [19], [20], [21], [22]. The JIS Z 2801 method entails the incubation of a microbial culture with the active material for 24 h after which serial dilutions and plate counts are used to determine the efficacy of the material. Though the method was found to yield repeatable results and truly reflects the surface antimicrobial activity of the material [15], each sample and organism tested requires multiple dilutions and plating for the numeration of viable cells which can become time and resource consuming.

Disk diffusion and strip diffusion antimicrobial assays are used in the field of antimicrobial solid surface development where either an existing material is chemically modified, or antimicrobial compounds are attached to or absorbed onto selected materials or surfaces. This assay serves as a quick and easy method to determine the optimal conditions and combinations to deliver the desired antimicrobial response. Diffusion assays are also common in pre-treatment testing in medicine to confirm which antibiotic will have the desired effect and would be a better option for treatment. In both applications, the disk diffusion assays are relatively easy to use but could become laborious when more than one antibiotic/organism needs to be tested [23]. The disk diffusion-type assays are also limited to slow-release and leaching materials modified with small polar/amphipathic compounds that will diffuse through the agar/agarose matrix to enable the clear halo formation in the microbial carpet or microcolony network [24,25]. Though both methods require very little infrastructure and training, it takes 20–48 h before results can be obtained.

**Fig. 1:**
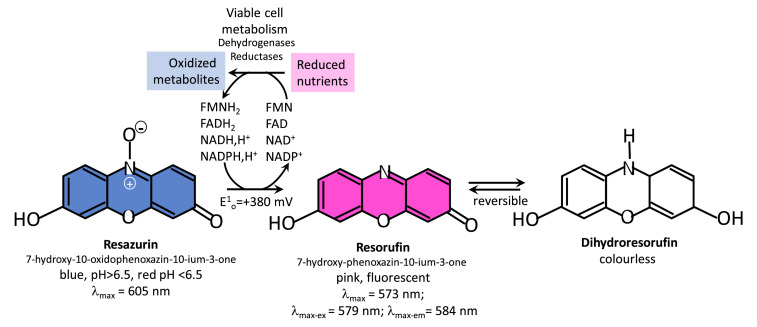
Conversion reactions of blue resazurin to its pink fluorescent counterpart, resorufin and colourless dihydroresorufin, upon reduction by reducing equivalents in metabolising/viable cells [26,32,33].

Resazurin-resorufin type assays utilise vitality/viability chemistry based on the redox status of the cells [26] (Fig. 1). Originally used to detect microbial infections in milk [27,28], it is now also used to determine the efficacy of novel drugs [24,25,29] or hormones [30,31], as well as their cytotoxicity [32,33]. Resazurin (7-hydroxy-10-oxidophenoxazin-10-ium-3-one) is proposed to be taken up by the cell and reduced by the mitochondrial enzymes transferring electrons from the appropriate reducing agent, such as NADH, FADH_2,_ FMNH_2_, NADPH and reduced cytochromes leading to the conversion to resorufin (7-hydroxy-3-phenoxazin-10-ium 3-one) (Fig. 1)[26,32,33]. Furthermore, the irreversible conversion of the resazurin to its fluorescent counterpart by reducing equivalents in cells, resorufin, can be directly correlated to the metabolic activity and vitality/viability of the target cells and by extrapolation, the number of viable cells. The high sensitivity of the dye lies with the increased conversion over time due to the continued cellular metabolism of the viable cells.

In this assay development, we adapted the JIS Z 2801 assay into 96-well format to make use of the smaller sample size and ease of disk diffusion and combined it with the detection sensitivity of resazurin dye into a high throughput hybrid assay to determine the solid surface activity of newly developed active materials. The two conventional assays and our adapted resazurin assay were evaluated by testing antibiotic-containing cellulose, as reference polymeric matrix, against four human bacterial pathogens *Escherichia coli, Pseudomonas aeruginosa, Staphylococcus aureus* and *Listeria monocytogenes*.

## Materials and methods

### Materials

Tetracycline was obtained from Boehringer Mannheim GmbH/Roche Diagnostics GmbH (Germany). Gentamicin, erythromycin, ampicillin, bacitracin, resazurin sodium salt and KCl were supplied by Sigma (St. Louis, MA, USA). Cellulose filters (Paper) (MN 615/No 1) were obtained from Macherey-Nagel (Düren, Germany). Acetonitrile, HPLC-grade far UV cut-off was supplied by Romil Ltd (Cambridge, UK). Merck (Darmstadt, Germany) supplied agar, yeast extract, tryptone, Na_2_HPO_4_, KH_2_PO_4_ and Merck (Wadeville, SA) supplied sodium chloride. Petri dishes were provided by Greiner bio-one (Frickenhausen, Germany). The 96-well polystyrene plates were acquired from Corning (Kennebunk, ME, USA) and Whatman Filter paper 1 from GE Healthcare Life Sciences supplied by Sigma-Aldrich (Darmstadt, Germany). Analytical grade water (milliQ water) was obtained by filtering water in a reverse osmosis plant through Millipore Milli-Q® water purification system (Milford, USA).

### Creating antibiotic discs as model self-sterilising surfaces/materials

The antibiotic-containing disks were utilised as antimicrobial or self-sterilising material models for a proof of concept study, therefore the antibiotic amounts used were not selected to mimic any therapeutic values. The selection of agents was chosen as representatives of possible active agents on solid surfaces. Amounts of 0.25 µg to 8.0 µg per active compound per cellulose disk (6 mm diameter) was used for the diffusion assay. Based on the results obtained, 1.0 µg of the respective compounds per disk was used for the JIS Z 2801 and resazurin assay. All the antibiotics were dissolved in water and disks prepared and dried in a 96-well plate to ease the process. Accurately assessing the contact killing ability of a novel solid surface would entail ensuring that leachable compounds are accounted for which can be achieved by washing the materials in water [15]. However, since the antibiotics were not actively attached to the cellulose, the materials were not washed before testing.

### Culturing of target organisms

A freezer stock of *Listeria monocytogenes* B73 was streaked out onto BHI agar plates (brain heart infusion) and incubated at 37 °C for 48 h until colonies were visible. *Escherichia coli* K12, *Staphylococcus aureus* RN 4220 and *Pseudomonas aeruginosa* ATCC 27853 were streaked out from freezer stocks onto LB agar plates (1% *m/v* NaCl, 1% *m/v* tryptone, 0.5% *m/v* yeast extract, 1.5% *m/v* agar in water) and incubated at 37°C for 24 h. Overnight starter cultures were made by selecting three to five colonies of the target organism and inoculating 1 mL of the respective growth medium. From the starter culture, a subculture was made into 6 mL of fresh media and grown until the mid-exponential growth phase was reached for each organism. All cultures were incubated at 37°C by shaking at 150 RPM at an angle. The mid-exponential growth phase and cell concentration for each organism was determined with classical plate counts as: *S. aureus* (OD = 0.30; ± 1.4 × 10^8^ cells/mL), *P. aeruginosa* (OD = 0.50; ± 2.9 × 10^8^ cells/mL), *L. monocytogenes* (OD = 0.40; 1.3 × 10^8^ cells/mL) and *E. coli* (OD = 0.40; 4 × 10^7^ cells/mL). Optical density (OD) was determined at 600 nm with a path length of 1.0 cm.

### Disk-diffusion antimicrobial assay

A culture of the target organism (1.0 mL) with 1 × 10^8^ cells/mL was used to cover each square pre-poured agar plate (10 × 10 cm) with a cotton tip. Once the plates were dried, the antibiotic-containing disks were transferred to the plate, incubated for 20 h and inspected for halo formation. NBT (nitroblue tetrazolium, 200 µg/mL in water) was incubated for 5 min with the *L. monocytogenes* plates to better visualize the halo formation. This visualisation was not deemed necessary for the other three organisms.

### Japanese industrial standard method JIS Z 2801

The assay was performed based on a standardised methodology [15,34,35]. A solid-surface sample with antibiotic was inoculated with 10 µL of a 1.4 × 10^7^ cells/mL cell culture, equivalent to the industrial standard of 5 × 10^5^ cells/cm^2^ and incubated for 24 h at 37°C in a humidity chamber to limit evaporation. Following the incubation, the material disk and incubated culture were transferred to a microcentrifuge tube and 1.00 mL of PBS added. The sample was vortexed for 60 s to dislodge any surviving cells from the surface after which the solution was serially diluted, plated onto agar plates and incubated at 30°C for 20–24 h. Based on the cell count the log reduction of cell number and thus the efficacy of the solid surface could be calculated by using the following equation:

$$Log\;reduction=log_{10}\;\frac{A}{B}$$

Where *A* is the number of CFU determined from the control sample and *B* the number of CFU determined from the antibiotic-containing materials. A cellulose disk containing no antibiotic was used as the growth control.

### Resazurin solid surface antimicrobial assay

The standard cell number used to challenge solid surface assays to confirm the antimicrobial activity is 5 × 10^5^ cells/cm^2^ with an inoculation volume of 10 µL to ensure surface activity [15]. Three cells concentrations were used for the resazurin assay: 5 × 10^4^ cells/cm^2^_,_ 5 × 10^5^ cells/cm^2^, 5 × 10^6^ cells/cm^2^. Mid-exponential growth cultures of the respective target organisms were diluted to the necessary cell concentration, transferred to a 96-well plate containing the antimicrobial disks and incubated for 1 h at 37°C. Antibiotic containing discs were placed in 96-well plates in triplicate for each cell concentration, including three controls for each of the antibiotics and three growth controls. After the hour incubation 90µL PBS (phosphate buffer saline: 0.8% *w/v* NaCl, 0.04% *w/v* KCl, 0.144% *w/v* Na_2_HPO_4_, 0.02% *w/v* KH_2_PO_4_; pH 7.4) and 10 µL resazurin was added to each well after which the plate was incubated at 37°C. Fluorescence readings were taken at Ex_530_ and Ems_590_ with the Tecan Spark 10M Multimode Microplate Reader and controlled by the Spark Control^TM^ software, both provided by Tecan Group Ltd (Mennedorf, Switzerland). The percentage inhibition, based on fluorescence (F), was calculated from the following calculation:

$$\%\;\mathrm{Inhibition}\;\mathrm{of}\;\mathrm{Target}\;\mathrm{organism}\;=\frac{F\;\mathrm{of}\;\mathrm{well}-F\;\mathrm{of}\;\mathrm{average}\;\mathrm{blank}}{F\;\mathrm{of}\;\mathrm{growth}\;\mathrm{control}-F\;\mathrm{of}\;\mathrm{average}\;\mathrm{blank}}$$

Readings were taken at time points selected for optimal resazurin conversion for each target organism, also considering the dye conversion versus cell number/cm^2^ (*supplementary data*). Furthermore, an arbitrary cut-off of 50% metabolic inhibition was used to determine if the material is classified as active (≥50% inhibition) or inactive (<50% inhibition). Due to the high cell number/cm^2^ used in the resazurin assay, this limit would still allow for the detection of cell numbers higher than that of the conventional 5 × 10^5^ cells/cm^2^ used in the JIS Z 2801 assay.

## Method validation

The study represents a proof-of-concept method for a range of solid surfaces with different active compounds thus the antibiotics were selected to represent a wide range of antimicrobial agents with different modes of action. The activity of each of the antibiotics was not the focus of the study, but they merely served as test compounds in the creation of model antimicrobial materials used to compare the three methods of activity determination. Five antibiotics were chosen to represent different classes, namely gentamicin (aminoglycoside), ampicillin (penicillin), bacitracin (cyclic branched peptide), gramicidin S (cyclic antibiotic peptide) and tetracycline (polyketide). They differ in size, chemistry (water solubility), mode of action and target(s). By extension, these differences influence the rate of activity and type of activity, which will highlight shortcomings within each of the two existing methods compared with our new method based on the ability of the assays to detect the activity of different antimicrobial compounds.

### Disk-diffusion

**Fig. 2:**
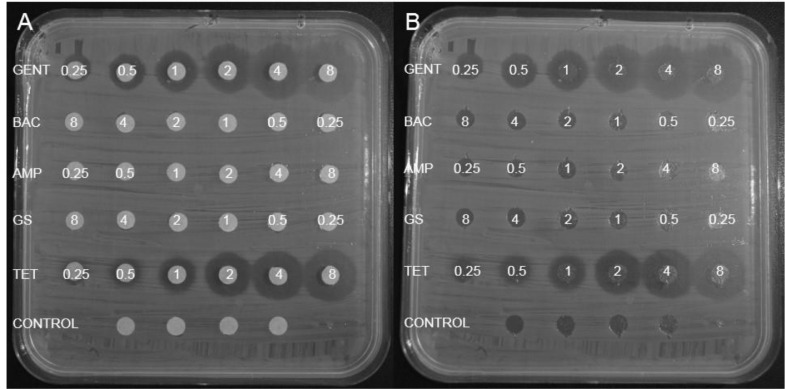
Examples of halo formation observed with disk diffusion, after 20 h of gentamicin (GENT), bacitracin (BAC), ampicillin (AMP), gramicidin S (GS), tetracycline (TET) against *E. coli***(A)** with and **(B)** with the disks removed. Note that the *E. coli* strain is known to be resistant to AMP, while GS and BAC has weak activity towards Gram negative bacteria.

Disk diffusion is probably one of the oldest methods used to determine the antimicrobial activity of new compounds [36,37] and relies on the diffusion of an active compound through a media agar plate, inhibiting the growth of the target microorganism around the disk or strip. Since the ability of the assay to detect microbially active materials is in question, activity will be defined as any halo formation regardless of size which would, however, indicate that the compound was no longer attached to the material. As some compounds cannot leach or diffuse in and through the agar, the disks were removed to inspect any microbial growth inhibition under the disks (Fig. 2:B, *supplementary data Figs. S1:B, S2:B, S3*). As the presence of the disks on the microbial lawn inhibits the growth of the target organism; detection of surface activity is limited to the formation of halos, and therefore only applicable to leaching compounds.

Gentamicin, ampicillin and tetracycline are all active against Gram-positive and Gram-negative bacteria, with the minimum inhibitory concentration (MIC) of each antibiotic varying depending on the target organism [38]. Gramicidin S has activity against both Gram-positive and Gram-negative bacteria, however, it loses all activity against Gram-negative bacteria in the presence of agar [39]. Bacitracin only has Gram-positive activity. This holds true for the activity against *E. coli* (Fig. 2), as an example: gentamicin and tetracycline show good activity and gramicidin S and bacitracin shows no activity, as expected. There is, however, detectable resistance of the *E. coli* strain in this study against ampicillin.

Based on the results obtained (Table 1, compiled from Fig. 2:A and *supplementary data Figs. S1:A, S2:A, S3)* the gentamicin containing material had the best overall activity, inhibiting all the target organisms at low amounts of the active agent. This was followed by the tetracycline containing material that inhibited the growth of all the organisms, except for *P. aeruginosa* for which it had a low level of activity. The gramicidin S containing material mainly worked against Gram-positive bacteria, whereas the bacitracin and ampicillin containing material had very little to no activity against the target organisms at the range of concentrations for the active agents tested. The conclusion would therefore be that the gentamicin and tetracycline containing material would hold promise for application, the GS containing material was either limited to applications against Gram-positive bacteria or in need of formulation with another active agent to combat Gram-negative colonisation. The bacitracin and ampicillin containing material would either be re-evaluated in terms of concentrations or be regarded as ineffective.

**Table 1:** Summary of the lowest halo-inducing concentration (µg per disk) for each of the antibiotic compounds against the selected organisms as determined by disk diffusion. The data represents the results from two independent repeats as μg compound needed to form a visible inhibition zone of at least 1 mm around the treated disk.

Compound/Organism	L. monocytogenes	S. aureus	E. coli	P. aeruginosa	Comments
Gentamicin	< 0.25	1	< 0.25	1	Broad spectrum of activity
Bacitracin	> 8	> 8	> 8	> 8	Not active
Ampicillin	> 8	8	> 8	> 8	Little activity against S. aureus
Gramicidin S	2	1	> 8	> 8	Only active against Gram-positive bacteria
Tetracycline	< 0.25	< 0.25	1	8	Broad spectrum activity except against P. aeruginosa

### Japanese industrial standard method

The Japanese Standard Assay JIS Z 2801 is a method used to determine the antimicrobial activity of solid surfaces, based on the count of viable cells after direct contact incubation with the surface. However, the method is resource and time-intensive and laborious when testing more than one organism and a variety of active matrices. As a result, only three antibiotic-containing materials were tested based on the results obtained between the disk diffusion assay and resazurin assay namely gramicidin S, gentamicin and bacitracin. Gentamicin proved to be active in both assays and served as the ‘positive control’ whereas bacitracin showed to have very little or no activity and served as the ‘negative control’. Gramicidin S showed very little activity against Gram-negative bacteria in the disk diffusion assay but had a much higher activity when tested with the resazurin assay (refer to the discussion later). Also, only *L. monocytogenes* and *P. aeruginosa* were selected as the target organisms because of their role in surface contamination in the health and food processing industries as well as both being human pathogens [2,5,40].

This method relies heavily on the successful detachment of single cells and subsequent CFU counts. Based on the observed cell counts the log reduction of each of the materials could be calculated and are summarised in Table 2. It is important to note that the log reduction reported for the control sample indicates the maximum possible number of cells killed by the active material, based on the cell count observed for the control sample. A cellulose disk containing no antibiotic was used as a control sample for full growth or survival.

**Table 2:** Comparison of the log reduction in cell counts (as log_10_ values) determined from the JIS Z 2801 assay to evaluate the activity of gramicidin S-, gentamicin- and bacitracin-containing solid surfaces (paper disks). Efficacy of the disks are expressed as a log reduction, with the control showing the highest possible log reduction as calculated from the full growth observed. A cellulose disk containing no antibiotic was used to determine the growth control. The data represents the mean log reduction ± standard deviation (SD) of three assays.

Compound/Organism	L. monocytogenes	P. aeruginosa	Comments
Gentamicin	6.4 ±0.1	6.4 ±1.3	Broad spectrum of activity
Bacitracin	0.8 ±0.3	No activity	Some activity against L. monocytogenes
Gramicidin S	5.6 ±1.2	No activity	Only active against L. monocytogenes
Control	6.4 ±0.1	7.4 ±0.1	Not applicable

The gentamicin-containing material could fully inhibit the growth of *L. monocytogenes* followed by gramicidin S-containing material (Table 2). The bacitracin-containing material did result in some activity, but it is not considered a viable option in terms of active material. Ideally, considering that the development of active materials aims to eradicate or control microbial infection, materials are expected to at least result in a log reduction of 1 (90% inhibition) of the target cell. Activity tests against *P. aeruginosa* showed only gentamicin having activity against the pathogen and resulting in full inhibition. No activity was observed for the bacitracin- and gramicidin S-containing materials.

Based on the results obtained, as summarized in Table 2, the gentamicin-containing material would hold the most promise in terms of preventing solid surface contamination. The gramicidin S-containing materials would also have some application even though it is just active against Gram-positive bacteria. Overall, the antibiotic-containing materials were deemed more active in general than determined by the disk diffusion assay where the recorded minimum inhibitory amount was 2 µg compared to the 1 µg tested with the JIS Z 2801 method.

### Resazurin solid surface antimicrobial assay

Resazurin as a microbial indicator was originally used in an assay developed for the detection of actively metabolising micro-organisms in contaminated milk [27,28]. The blue dye is reduced by the cells to its pink/red fluorescent counterpart, resorufin, but can be further reduced to dihydroresorufin which is translucent and non-fluorescent [32]. The reduction of the dye can be caused by sugars in media and certain antibiotics with reducing character, as was observed in this study at high concentrations of tetracycline. The dye also auto-reduces in light, and therefore the maximum suggested time for incubation with the dye is four hours. Other than the controls of media and solid surfaces, the conversion of resazurin by the organism and resazurin concentration should be optimised to ensure the best results possible which was done for all the target organisms in this study (supplementary data). As in choosing the best incubation times, it is imperative to select a time point that is sensitive to the lowest cells/cm^2^ without sacrificing the linearity of detection. The active materials were incubated at 5 × 10^5^ cells/cm^2^ (which is the industry standard) and 5 × 10^6^ cells/cm^2^ (which is 10-fold more than the standard). An optimum incubation time was selected for each of the cells counts for each of the organisms. It was observed that the cellulose paper alone did inhibit some growth leading to slightly lower conversion and resorufin fluorescence detected. However, incubation time points were still selected based on the original growth curves in the absence of the cellulose matrix. Furthermore, with some antibiotics greater than 100% inhibition was reported. This is possibly due to entrapment of the resazurin either within cells at low cell numbers or within the cell debris and cellulose matrix. Such cases were considered as full inhibition of the target organism.

**Fig. 3.**
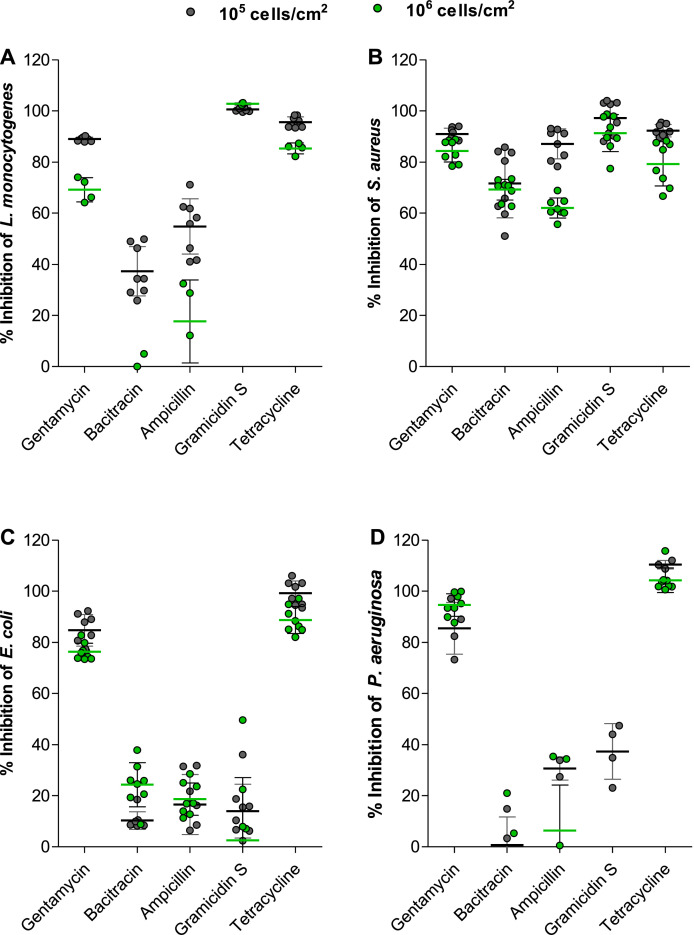
Percentage inhibition of 1.0 µg active compound against **(a)***L. monocytogenes***(b)***S. aureus***(c)***E. coli* and **(d)***P. aeruginosa* at 5 × 10^5^ and 5 × 10^6^ cells/cm^2^. The data bars are representative of the mean and SD of 8 repeats for (A–C) and 4 repeats for (D).

The incubation time selected for *L monocytogenes* based on the best resazurin conversion was at 30 min and 1 h for 5 × 10^6^ cells/cm^2^ and 5 × 10^5^ cells/cm^2^ respectively (Fig. 3: A). Incubation time points selected for *S. aureus* and *E. coli* were at 1 h and 2 h for 5 × 10^6^ cells/cm^2^ and 5 × 10^5^ cells/cm^2^ respectively (Fig. 3: B and C). The incubation time point selected for *P. aeruginosa* was at 4 h after incubation for both cell counts due to the slow conversion of resazurin by the organism (Fig. 3: D).

Overall, the activity of the antibiotics decreased with an increase in target cell number as expected. The only exception is seen for bacitracin, ampicillin and gramicidin S against *E. coli* which, based on the disk diffusion assays, can be regarded as inactive. We defined inactive as metabolic inhibition that is less than 50% (for more details refer to *supplementary data text and Figs. S4 and S5*). In terms of assessing the overall activity of each of the materials (Fig. 3, summarised in Table 3), gentamicin- and tetracycline-containing materials have good broad-spectrum activity, inhibiting either 90% or 100% of the target organism metabolism. The gramicidin S-material, at the amount that it was tested, only exhibited activity against the Gram-positive bacteria *L. monocytogenes* and *S. aureus* (Fig. 3). Furthermore, ampicillin-materials did not have activity against the penicillin-resistant *E. coli*, presented mainly Gram-positive activity and is more effective against inhibiting *S. aureus.* Bacitracin-material overall exhibited very low activity at the amount that it was tested with some activity against *S. aureus* and *L. monocytogenes*. Thus, the activity of the materials can be ranked from most effective to least effective as gentamicin/tetracycline> gramicidin S> ampicillin> bacitracin.

**Table 3:** Comparison of the percentage growth/metabolic inhibition of each of target organisms at a cell count of 5 × 10^5^ cells/cm^2^ by the active materials. The data is representative of 8 repeats with the mean ± SD.

Compound/Organism	L. monocytogenes	S. aureus	E. coli	P. aeruginosa	Comments
Gentamicin	89 ±0.8	91 ±2.3	85 ±6.2	85 ±10	Broad spectrum activity
Bacitracin	37 ±9.6	72 ±13	10 ±3.4	0 ±15	Some activity against S. aureus and L. monocytogenes
Ampicillin	55 ±11	87 ±5.9	17 ±12	11 ±19	Some activity against S. aureus and L. monocytogenes
Gramicidin S	100 ±0.8	97 ±6.7	14 ±10	5.6 ±35	Gram-positive activity
Tetracycline	96 ±2.1	92 ±2.4	99 ±4.8	110 ±1.6	Broad spectrum of activity

### Assay comparison

The widespread effect of surface colonization and subsequent biofilm formation, product spoilage or patient infection is a global issue. Much focus is placed on the development of new and better surfaces to prevent adhesion all together. However, current methods to screen these active materials are lacking in that they are time-consuming, costly and laborious which ultimately limits material development and screening. We set out to compare disk diffusion, a Japanese industrial standard for solid surface activity detection and our new resazurin containing method. Disk diffusion and the resazurin assay were evaluated with four target organisms (*E. coli, P. aeruginosa, S. aureus* and *L. monocytogenes*) and five active compounds (gentamicin, bacitracin, ampicillin, gramicidin S and tetracycline). The effectiveness of each assay was determined by both comparing the observed sensitivity of the assays to detect the activity of the active polymers and the technical aspects of each of the assays. As each method makes use of different cell concentrations, the methods could not be directly compared in terms of detected mortality of cells/cm^2^. The results are summarised and compared in Table 4, in terms of whether the surfaces would have been considered effective by each respective method. All three methods yielded similar results, except for bacitracin and ampicillin having some activity against *S. aureus* and *L. monocytogenes*. Therefore, the novel resazurin assay can be interchanged with either disk diffusion or JIS Z 2801 in the screening for antimicrobially active materials.

**Table 4.** Comparison of the results for each of the active surfaces as determined by the different solid surface assays.

Compound/Assay	Disk diffusion	JIS Z 2801	Resazurin Assay
Gentamicin	Broad spectrum activity	Broad spectrum activity	Broad spectrum activity
Bacitracin	Not active	Some activity against *L. monocytogenes*	Some activity against S. aureus and L. monocytogenes
Ampicillin	Little activity against S. aureus	Not tested	Some activity against S. aureus and L. monocytogenes
Gramicidin S	Gram-positive activity	Gram-positive activity	Gram-positive activity
Tetracycline	Broad spectrum activity except against P. aeruginosa	Not tested	Broad spectrum of activity

Regarding methodology (Table 5), the disk diffusion assay has the benefit of being a fully optimized method with clear activity guidelines/thresholds for a range of organisms and compounds that requires very little training and infrastructure. However, this assay cannot be used to accurately assess the activity of contact killing on surfaces but is more suited to assess slow-release and leaching of antimicrobial compounds from treated materials. Admittedly, the current study did not select for non-leaching materials since the active compounds were not covalently attached to the cellulose disks. Both conventional assays gave similar results regarding the activity of the active materials, but results could be obtained with the resazurin assay within 3–8 h after incubation. In the JIS Z 2801, the activity of the solid surfaces is determined through CFU counts which is a laborious manual exercise. This is overcome in the resazurin assay by directly correlating cell metabolism to the number of viable cells on the surface of the material that is then converted to the effectivity of the active material. The assay is also performed in a 96-well plate which enables a broader range of materials and target cells to be tested with a larger number of controls and technical repeats. This high-throughput adaptation allows for quicker screening of materials with fewer consumables used, ultimately resulting in a similar assay to JIS Z 2801, but cheaper and faster. Moreover, due to the assay being performed in a 96-well plate it can be easily automated by using a robot dispenser. Our resazurin assay can easily be converted to larger format/volume vessels to test larger surfaces, but at a lower throughput. The presence of an active compound can lead to cell stress and consequential increased catabolism, opposed to cell death. This would lead to a false inhibition result due to the over conversion of pink/fluorescent resorufin to colourless dihydroresorufin (refer to Fig. 1) compared to the slower conversion to resorufin in the growth control. The activity of a material should strictly be expressed as the decreased conversion of resazurin which is due to lower cell metabolism. It is also feasible to measure the activity of these samples at earlier time points to still assess the resorufin before conversion to dihydroresorufin. Should false inhibitory results be observed (colourless result or lack of blue colour), materials could still be useful in antimicrobial treatments and could be tested for other functionalities such as biofilm prevention or used in combination with other active compounds or treatments.

**Table 5:** Comparative summary of advantages, disadvantages and time to results of the disk diffusion, JIS Z 2801 and resazurin assay.

	Disk Diffusion	JIS Z 2801	Resazurin Assay
Advantages	- Established method, requires no optimisation - Inexpensive - Requires little instrumentation - Medium throughput - Requires little sample	- True measure of solid surface activity - Direct contact with cells - Not limited by type of active compound tested	- True measure of solid surface activity - Direct contact with cells - Not limited by type of active compound tested - Can test different cell concentrations - High throughput - Requires little sample
Disadvantages	- Limited to active compounds that can diffuse through agar - Limited to slow-release materials	- Limited by effectivity of removing cells from surface and CFU plate count - Laborious and costly - Not high throughput - Requires large amounts of sample	- Need to determine conversion of resazurin by target organism - Cell stress can cause false results
Time from incubation to results	20–24 h	40–48 h	3–8 h

## Conclusion

We optimised a method containing resazurin to determine the antimicrobial activity of an active solid surface. This method yields similar results as an industrial standard method, JIS Z 2801, in a shorter time frame and at lower cost. Due to the use of a 96-well plate, the assay is high throughput and can be used to screen multiple samples at a time. This would enable the testing of multiple parameters within the creation of antimicrobial materials and surfaces. The use of higher cell counts (ten times higher) than the standard of 5 × 10^5^ cells/cm^2^ allows for faster detection of inhibition but more importantly selects for highly active materials. Furthermore, it can be used as a substitute for the disk diffusion assay in the medical sector where the assay can determine the suitability of antibiotics for treatment. The assay makes use of both disk diffusion and broth assay methodologies and can therefore be adapted to low-cost sterility testing in environments that have low infrastructure.

## Declaration of Competing Interest

The authors declare that they have no known competing financial interests or personal relationships that could have appeared to influence the work reported in this paper.

## Appendix Supplementary materials

Image, application 1
