# Supplementary material for: High throughput method to determine the surface activity of antimicrobial polymeric materials
Source: MethodsX. 2021 Nov 25;8:101593. doi: 10.1016/j.mex.2021.101593 (PMC8720914; doi:10.1016/j.mex.2021.101593)
Supplement: Supplementary file 1 [file mmc1.docx]

# SUPPLEMENTARY DATA

# High throughput method to determine the surface activity of antimicrobial polymeric materials

**Supplementary data**

Van Rensburg, W ; Laubscher, WE; Rautenbach, M^*^

BIOPEP^TM^ Peptide Group, Department of Biochemistry, Faculty of Science University of Stellenbosch, South Africa, E-mail: [mra@sun.ac.za](mailto:mra@sun.ac.za)

Disk diffusion

Examples of the disk diffusion results for *P. aeruginosa* (Figure S1), *S. aureus* (Figure S2) and *L. monocytogenes* (Figure S3) are given below.


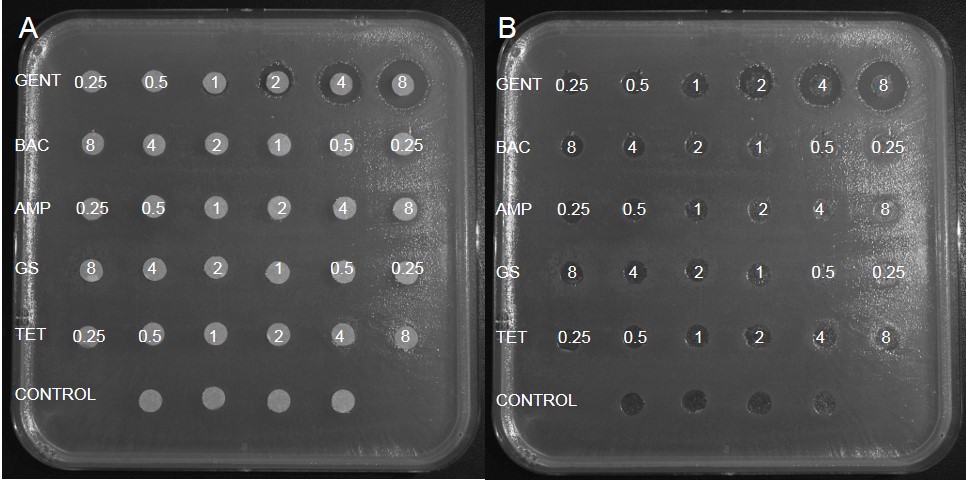


**Figure S1** An example of halo formation observed with disk diffusion, after 20 hours of gentamicin (GENT), bacitracin (BAC), ampicillin (AMP), gramicidin S (GS), tetracycline (TET) against *P. aeruginosa* **(A)** with and **(B)** without the disks. Note that only activity is observed for gentamicin.


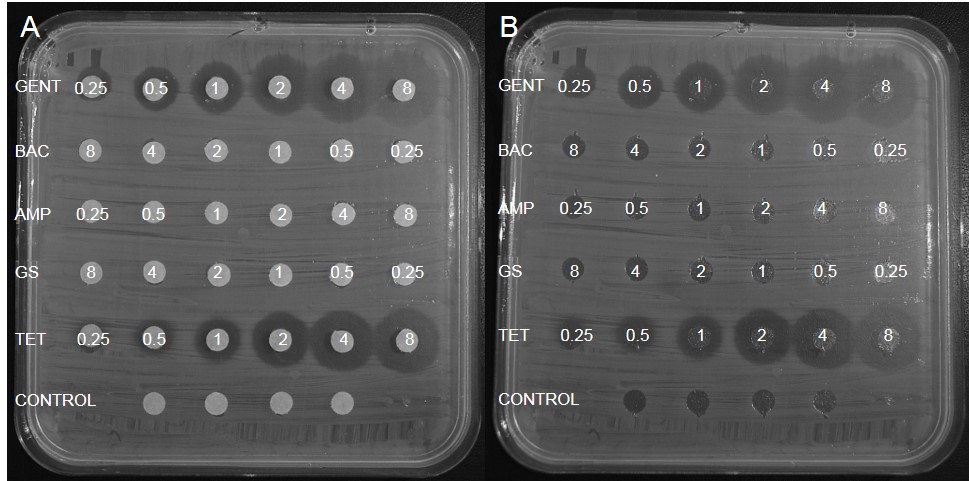


**Figure S2** An example of halo formation observed with disk diffusion, after 20 hours of gentamicin (GENT), bacitracin (BAC), ampicillin (AMP), gramicidin S (GS), tetracycline (TET) against *S. aureus* **(A)** with and **(B)** without the disks. Note the activity of gentamicin and tetracycline.


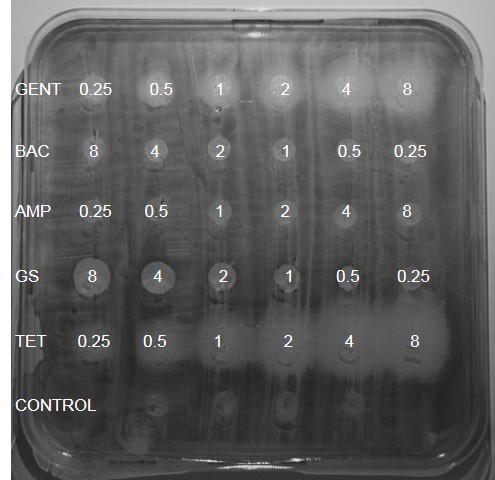


**Figure S3** An example of halo formation observed with disk diffusion, after 20 hours of gentamicin (GENT), bacitracin (BAC), ampicillin (AMP), gramicidin S (GS), tetracycline (TET) against *L. monocytogenes* without the disks. Halo visualisation was dependent on the NBT conversion of *L. monocytogenes.* Note the activity of gentamicin, gramicidin S and tetracycline.

Optimisation of the resazurin solid surface assay

Resazurin is used to detect the activity of compounds by directly correlating the amount of resorufin (fluorescent counterpart of resazurin) to the metabolism of viable cells. It is therefore first important to determine the linearity of the dye conversion to cell number to fully ascertain the percentage of inhibition caused by the active compound. Secondly, to determine the repeatability between cultures in terms of lowest detectable cell count within four hours which will ultimately affect the sensitivity of the assay. Lastly, a arbitrary cut-off of 50% metabolic inhibition was used to determine if the material is classified as active (≥50% inhibition) or inactive (<50% inhibition). The target cells were cultured as previously described, a dilution of cells incubated with three concentrations of resazurin, and the fluorescence read every hour for four hours. Due to the fast conversion of resazurin by *L. monocytogenes* readings were taken at 30 minutes and then every hour. Conversion of cells/mL to cells/cm^2^ was done by calculating the number of cells added to each well in 10 µL culture divided by the surface area of the well/disk (0.28cm^2^).

Incubation of *E. coli* showed good linearity after one hour of incubation for all three concentrations of resazurin (Fig. S4), with the lowest cell number detected as 10^4^ cells/cm^2^. The presence of 10^3^ cells/cm^2^ can be confirmed after 4 hours, but due to the maximum dye conversion at the growth control this could only be confirmed as growth or as not full inhibition. A study of the conversion curves at the higher cell numbers at three and four hours of incubation shows the plateau that forms due to the maximum dye that is converted. When comparing the concentrations of resazurin, it is clear the 0.03 mg/mL solution (Fig. S4: A) is too sensitive to changes in cell metabolism and could very easily lead to false conclusions. The results for 0.3 mg/mL and 3.0 mg/mL (Fig. S4: B and C, respectively) showed similar results in repeatability, however 0.3 mg/mL is more sensitive in the amount of relative fluorescence units (RFU) detected. This is crucial to better distinguish between cell metabolism, by extension cell counts and ultimately percentage inhibition when in contact with the active materials. Similar results were observed with *S. aureus* (Fig. S5): 0.03 mg/mL yields similar results, but 0.3 mg/mL is more sensitive in RFU. The best incubation time to read the fluorescence is at one hour due to the linearity of cells/cm^2^ and detected fluorescence. The lowest confirmed cell count is at 10^4^ cells/cm^2^ after one hour which could not be improved with a longer incubation time. The fluorescence detected at 3.0 mg/mL for all the organisms were similar to the low fluorescence at 0.03 mg/mL. The low fluorescence detected at 3.0 mg/ml resazurin is possibly due to masking of the resorufin (pink) by the excess resazurin (blue) whereas the low fluorescence detected 0.03 mg/mL resazurin is possibly a combination between low amount of resorufin formed and the formation of the non-fluorescent dihydroresorufin.

**Figure S4** Fluorescence observed for a dilution of *E. coli* cells over four hours at resazurin concentrations of **(A)** 0.03mg/mL **(B)** 0.3mg/mL and **(C)** 3mg/mL. The data points represent the mean of three technical and three biological repeats with error bars showing the standard error of the mean (SE).

**Figure S5** Fluorescence observed for a dilution of *S. aureus* cells over four hours at resazurin concentrations of **(A)** 0.03mg/mL **(B)** 0.3mg/mL and **(C)** 3mg/mL. The data points represent the mean of three technical and three biological repeats with error bars showing the standard SE.

As previously mentioned, *L. monocytogenes* converts the resazurin very quickly giving the best growth curve in terms of cell concentration to conversion at 30 minutes of incubation time for each of the dye concentrations (Fig. S6). Regarding resazurin concentrations, 0.03 mg/mL is too sensitive and results in inconsistent growth curves. The higher 3.0 mg/mL resazurin has good repeatability between biological repeats but is not sensitive enough to distinguish between the different concentrations with the lowest detection being 10^3^ cells/cm^2^ after 3 hours. The best concentration of resazurin is 0.3 mg/mL since it gives both a good linearity at 30 minutes incubation, is sensitive enough to detect the range of cell concentrations and can confirm the presence of 10 cells/cm^2^ after 3 hours of incubation.

**Figure S6** Fluorescence observed for a dilution of *L. monocytogenes* cells over three hours at resazurin concentrations of **(A)** 0.03mg/mL **(B)** 0.3mg/mL and **(C)** 3mg/mL. The data points represent the mean of three technical and three biological repeats with error bars showing the standard SE.

*P. aeruginosa* converts resazurin very slowly (Fig. S7), even with the low concentration 0.03 mg/mL resazurin gives low fluorescence results. All three concentrations of resazurin gives the same trend in cell number to fluorescence and can only detect 10^4^ cells/cm^2^ after 4 hours of incubation. Based on the sensitivity and highest fluorescence signal, 0.3mg/mL resazurin gave the best results.

**Figure S7**  Fluorescence observed for a dilution of *P. aeruginosa* cells over four hours at resazurin concentrations of **(A)** 0.03mg/mL **(B)** 0.3mg/mL and **(C)** 3mg/mL. The data points represent the mean of three technical and three biological repeats with error bars showing the standard SE.
